# Differential levels of Neurofilament Light protein in cerebrospinal fluid in patients with a wide range of neurodegenerative disorders

**DOI:** 10.1038/s41598-020-66090-x

**Published:** 2020-06-08

**Authors:** C. Delaby, D. Alcolea, M. Carmona-Iragui, I. Illán-Gala, E. Morenas-Rodríguez, I. Barroeta, M. Altuna, T. Estellés, M. Santos-Santos, J. Turon-Sans, L. Muñoz, R. Ribosa-Nogué, I. Sala-Matavera, B. Sánchez-Saudinos, A. Subirana, L. Videla, B. Benejam, S. Sirisi, S. Lehmann, O. Belbin, J. Clarimon, R. Blesa, J. Pagonabarraga, R. Rojas-Garcia, J. Fortea, A. Lleó

**Affiliations:** 1 Université de Montpellier, CHU de Montpellier, Laboratoire de Biochimie-Protéomique clinique, INSERM U1183 Montpellier, France; 2 Department of Neurology, Sant Pau Memory Unit, Hospital de la Santa Creu i Sant Pau - IIB Sant Pau, Barcelona, Spain; 3 Centro de Investigación Biomédica en Red en Enfermedades Neurodegenerativas, Ciberned, Spain; 4 Barcelona Down Medical Center, Fundació Catalana Síndrome de Down, Barcelona, Spain; 5 Department of Neurology, Neuromuscular Diseases Unit, MND Clinic, Hospital de la Santa Creu i Sant Pau, Biomedical Research Institute Sant Pau, Universitat Autònoma de Barcelona, Barcelona, Spain; 6 Centro de Investigación Biomédica en Red en Enfermedades Raras, Ciberer, Spain; 7 Department of Neurology, Movement Disorders Unit, Hospital de la Santa Creu i Sant Pau - IIB Sant Pau, Barcelona, Spain

**Keywords:** Biomarkers, Neurology

## Abstract

Cerebrospinal fluid (CSF) biomarkers are useful in the diagnosis and the prediction of progression of several neurodegenerative diseases. Among them, CSF neurofilament light (NfL) protein has particular interest, as its levels reflect neuroaxonal degeneration, a common feature in various neurodegenerative diseases. In the present study, we analyzed NfL levels in the CSF of 535 participants of the SPIN (Sant Pau Initiative on Neurodegeneration) cohort including cognitively normal participants, patients with Alzheimer disease (AD), Down syndrome (DS), frontotemporal dementia (FTD), amyotrophic lateral sclerosis (ALS), dementia with Lewy bodies (DLB), progressive supranuclear palsy (PSP) and corticobasal syndrome (CBS). We evaluated the differences in CSF NfL accross groups and its association with other CSF biomarkers and with cognitive scales. All neurogenerative diseases showed increased levels of CSF NfL, with the highest levels in patients with ALS, FTD, CBS and PSP. Furthermore, we found an association of CSF NfL levels with cognitive impairment in patients within the AD and FTD spectrum and with AD pathology in DLB and DS patients. These results have implications for the use of NfL as a marker in neurodegenerative diseases.

## Introduction

Biomarkers of neurodegenerative diseases are key for the evaluation, differential diagnosis and follow-up of patients with cognitive impairment or dementia. In particular, three cerebrospinal fluid (CSF) biomarkers (β-amyloid 1–42 [Aβ_1–42_], total Tau [t-Tau] and its phosphorylated form [p-Tau]) have been extensively studied due to their high diagnostic accuracy for the diagnosis of Alzheimer disease (AD)^1^. Thus, the quantification of these biomarkers in the CSF is currently being implemented in clinical practice either to confirm the biochemical AD signature in the evaluation of a patient with mild cognitive impairment (MCI) or dementia or to exclude it in other dementia syndromes, such as frontotemporal dementia (FTD) or dementia with Lewy Bodies (DLB)^2,3^. More recently, other CSF biomarkers, such as YKL-40, the soluble β fragment of amyloid precursor protein (sAPPβ), neurogranin, glial fibrillary acidic protein (GFAP) or Neurofilament Light (NfL), have been described to be potentially informative for the discrimination of various neurodegenerative conditions, such as AD, FTD, progressive supranuclear palsy (PSP), corticobasal syndrome (CBS), or DLB^4–8^.

Neurofilaments provide structural suport to neurons. Different forms of neurofilaments exist, including NfL, which is strongly expressed in myelinated axons and physiologically secreted in small amounts in the CSF. Disruption of neurofilament organisation is one of the key characteristics of many neurological conditions, such as amyotrophic lateral sclerosis (ALS), AD, FTD or vascular dementia among others^6,9,10^. In addition, recent studies have shown that levels of NfL in CSF are associated with clinical progression and severity in ALS^11–13^ and in other neurodegenerative diseases due to its capacity to reflect the extent of neuro-axonal damage^14,15^. Recently, a large control-case study that included various neurodegenerative disorders confimed the importance of CSF NfL in the evaluation and follow-up of patients with cognitive impairment^16^: in particular, CSF NfL levels were increased in patients with a diagnosis of MCI, AD, FTD or ALS compared to controls, thus reflecting the intensity of neurodegenerative processes.

In the present work, we investigated CSF NfL in the SPIN cohort^17^, which includes a variety of neurodegenerative disorders, such as AD, FTD, ALS, DLB, PSP, CBS and subjects with Down syndrome (DS). This is the first time to our knowledge that such different phenotypes can be compared through a monocentric cohort. We compared CSF NfL levels accross these disorders and studied its association with other CSF biomarkers, with the severity of cognitive impairment and with the presence of AD pathology in DLB (DLB-AD) and DS (DS-AD). This study highlights the potential role of CSF NfL for the early diagnosis (including prodromal stages) and follow-up of DLB patients.

## Results

### Demographics and core CSF biomarkers

We included a total of 535 participants from the SPIN cohort, comprising 118 cognitively healthy participants and 417 patients with various neurodegenerative disorders (Table 1). Age was different among the groups (F = 79.438, p < 0.001) but there was no significant difference in the male:female ratio. As expected, frequency of *APOE*ε4 allele was significantly higher in AD patients than in the other groups (X^2^ = 52.7, p = 0.001), and no differences were observed among the other groups. As expected, MMSE scores were lower in all clinical groups compared to control subjects (F = 11.972, p < 0.001).

**Table 1:** Demographic and clinical data, cognitive scores, *APOE*e4 status and CSF biomarker concentrations of all participants.

Diagnosis	Total patients (n)	Age mean (SD)	Total women (%)	Patients taking MMSE (n)	MMSE score mean (SD)	*APOE*ε 4 allele (%)	Median (interquartile range)			
							NfL	Aβ_1–42_	Total Tau	p-Tau
Control	118	59.4 (9.7)	68 (58%)	117	29.1 (1.0)	32 (28%)	411 (343–567)	818 (648–991)	205 (152–260)	39 (34–51)
Alzheimer disease	116	70.4 (8.0)	71 (61%)	111	22.9 (4.8)	65 (56%)	940 (765–1229)	414 (330–484)	631 (466–874)	88 (73–108)
Down syndrome	47	37.2 (9.4)	20 (43%)	0	NA	10 (21%)	349 (196–464)	754 (545–921)	172 (101–254)	33 (22–58)
Down syndrome with Alzheimer disease	50	51.2 (8.1)	21 (42%)	1	NA	8 (16%)	955 (664–1497)	413 (333–461)	520 (245–1008)	77 (45–124)
Amyotrophic lateral sclerosis	46	65.6 (11.3)	22 (48%)	46	20.2 (14.0)	NA	2953 (1664–4250)	350 (254–555)	313 (242–461)	42 (33–52)
Frontotemporal dementia	56	65.8 (5.2)	15 (27%)	56	23.9 (7.2)	12 (21%)	1240 (859–2378)	739 (540–941)	229 (188–338)	41 (29–56)
Dementia with Lewy bodies	37	76.7 (4.9)	19 (51%)	36	22.1 (4.3)	12 (32%)	1135 (803–1321)	539 (428–752)	326 (219–659)	54 (42–93)
Prodromal Dementia with Lewy bodies	26	82.2 (6.1)	13 (50%)	26	25.8 (2.6)	8 (31%)	934 (643–1094)	523 (496–862)	307 (226–473)	54 (47–78)
Corticobasal syndrome	26	72.0 (7.3)	13 (50%)	22	22.5 (5.3)	5 (23%)	1637 (923–2797)	696 (479–911)	302 (209–424)	51 (40–64)
Progressive supranuclear palsy	12	70.5 (7.8)	7 (58%)	10	26.0 (3.7)	2 (17%)	1422 (1034–1727)	664 (426–879)	219 (157–309)	36 (30–43)

Abbreviations: MMSE: Mini-Mental State Examination, NA: Not Applicable, NfL: Neurofilament Light.

There were differences in CSF core AD biomarkers (Aβ_1–42_, t-Tau and p-Tau) among the groups (Table 1). In particular, Aβ_1–42_ was significantly lower in all groups (F = 30.551, p < 0.001) compared to control subjects, Table 1. T-Tau was significantly increased in all groups except in DS (F = 26.863, p < 0.001) compared to control participants, Table 1. Levels of p-Tau were significantly increased in AD, DS-AD, DLB, prodDLB and CBS groups (F = 24.079, p < 0.001) compared to control participants, Table 1.

### Relationship between CSF NfL and age, gender, cognitive scores or core AD biomarkers

CSF NfL levels were positively correlated with age (ρ = 0.490, p < 0.001) and were associated with sex (higher in males, t = 2.592, p = 0.01) in the entire cohort. All group comparisons and correlation analysis were thus subsequently age and sex-adjusted. In addition, as shown in Table 2, CSF NfL levels negatively correlated with Mini-Mental State Examination (MMSE) scores in control participants, AD, prodDLB and FTD groups. CSF NfL levels positively correlated with t-Tau levels in control participants, AD, ALS, DS and DS-AD. NfL and p-Tau levels were positively correlated in control participants, AD, DS and DS-AD. Aβ_1–42_ levels were negatively correlated with NfL in FTD group.

**Table 2:** Correlation between CSF NfL and MMSE or core biomarkers among the clinical groups.

Diagnosis	CSF NfL-MMSE correlation	CSF NfL-total Tau correlation	CSF NfL-pTau correlation	CSF NfL-Aβ_1–42_ correlation
Control	ρ= −0.202 (p = 0.030)	ρ = 0.500 (p < 0.001)	ρ = 0.522 (p < 0.001)	ρ = 0.119 NS
Alzheimer disease	ρ = −0.188 (p = 0.045)	ρ= 0.363 (p < 0.001)	ρ= 0.380 (p < 0.001)	ρ = 0.026 NS
Down syndrome	NA	ρ = 0.692 (p< 0.001)	ρ= 0.667 (p < 0.001)	ρ = −0.103 NS
Down syndrome with Alzheimer disease	NA	ρ = 0.755 (p< 0.001)	ρ = 0.702 (p< 0.001)	ρ = −0.288 NS
Amyotrophic lateral sclerosis	ρ= −0.047 NS	ρ= 0.350 (p = 0.025)	ρ = 0.059 NS	ρ = 0.182 NS
Frontotemporal dementia	ρ = −0.345 (p= 0.010)	ρ = 0.121 NS	ρ = −0.117 NS	ρ= −0.329 (p = 0.020)
Dementia with Lewy bodies	ρ = −0.254 NS	ρ = 0.313 NS	ρ = 0.114 NS	ρ = 0.059 NS
Prodromal Dementia with Lewy bodies	ρ= −0.431 (p = 0.039)	ρ = 0.473 NS	ρ = 0.270 NS	ρ = 0.313 NS
Corticobasal syndrome	ρ = −0.202 NS	ρ = 0.321 NS	ρ = 0.293 NS	ρ = 0.348 NS
Progressive supranuclear palsy	ρ= −0.268 NS	ρ = 0.400 NS	ρ = 0.444 NS	ρ = −0.133 NS

Abbreviations: MMSE: Mini-Mental state examination, NS: not statistically significant, NA: Not Applicable, NfL: Neurofilament Light.

### CSF NfL levels accross clinical groups

**Figure 1:**
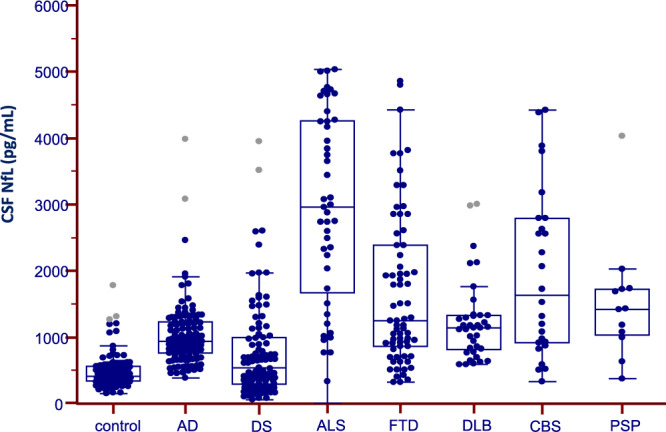
CSF Neurofilament Light (NfL) protein levels in the SPIN cohort. Box and whisker plots of the median concentrations of CSF NfL in control participants and patients with Alzheimer Disease (AD), Down Syndrome (DS), Dementia with Lewy Bodies (DLB), Amyotrophic Lateral Sclerosis (ALS), Frontotemporal Dementia (FTD), Corticobasal Syndrome (CBS) and Progressive Supranuclear Palsy (PSP). The central black lines show the median values, regions above and below these lines show the upper and lower quartiles, respectively. Outliers (indicated with grey circles) are defined as a value that is larger than the upper quartile plus three times the interquartile range.

CSF NfL levels were elevated in all groups (with exception of DS) compared to control participants (F = 40.809, p < 0.001), Table 1 and Fig. 1.

#### FTLD-related clinical syndromes (ALS, FTD, CBS, PSP)

**Figure 2:**
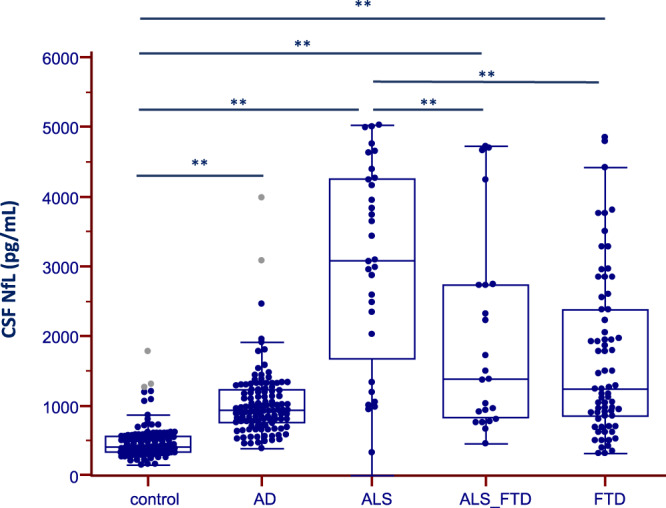
CSF Neurofilament light (NfL) protein levels in patients within the FTD-ALS spectrum. Box and whisker plots of the median concentrations of CSF NfL in control participants and patients with dementia: Alzheimer Disease (AD) and Amyotrophic Lateral Sclerosis associated or not with FTD (ALS-FTD and ALS, respectively) and Frontotemporal Dementia (FTD). The central black lines show the median values, regions above and below these lines show the upper and lower quartiles, respectively. Outliers (indicated with grey circles) are defined as a value that is larger than the upper quartile plus three times the interquartile range. **p = 0.005.

The highest CSF NfL levels were found in the ALS group, followed by patients with CBS, PSP and FTD (Table 1 and Fig. 1). All these groups showed higher CSF NfL levels compared to control participants (p < 0.001), Table 1*.* We found a gradient in NfL levels in the ALS-FTD spectrum (Fig. 2) with highest levels in patients with ALS without FTD (median = 3093, IQR = [2107–4261] pg/mL) followed by those with ALS-FTD (median = 1386, IQR = [836–2731] pg/mL) p = 0.005, and those with FTD without motor neuron symptoms (median = 1240, IQR = [859–2378] pg/mL). The AUC for CSF NfL for the detection of ALS in patients with FTD was 0.705 (95% CI 0.576–0.874).

#### DLB patients

**Figure 3:**
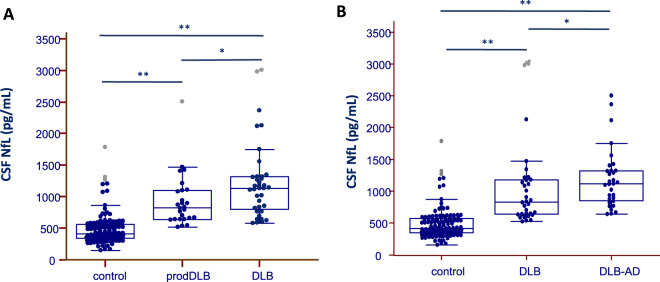
CSF Neurofilament light (NfL) protein levels in patients with Dementia with Lewy Bodies (DLB). (**A**) Box and whisker plots of the median concentrations of CSF NfL in control participants and patients with Dementia with Lewy Bodies (DLB) or prodromal DLB (prodDLB). The central black lines show the median values, regions above and below these lines show the upper and lower quartiles, respectively. Outliers (indicated with grey circles) are defined as a value that is larger than the upper quartile plus three times the interquartile range. (**B**) Box and whisker plots of the median concentrations of CSF NfL in control participants and patients with Dementia with Lewy Bodies (DLB) and DLB with AD pathology AD (DLB-AD). The central black lines show the median values, regions above and below these lines show the upper and lower quartiles, respectively. Outliers (indicated with grey circles) are defined as a value that is larger than the upper quartile plus three times the interquartile range. *p < 0.05, **p < 0.001.

The DLB group showed higher CSF NfL levels compared to control participants (p < 0.001), Table 1 and Fig. 1. Interestingly, when comparing prodDLB and DLB patients, we observed a significant and gradual increase in CSF NfL levels in these subgroups (p = 0.01), Fig. 3A, while the levels of the core AD biomarkers (t-Tau, p-Tau and Aβ_1–42_) were comparable (data not shown). The AUC for CSF NfL comparing prodDLB and DLB patients was 0.694 (95% CI 0.564–0.805). Compared to control subjects, CSF NfL was increased in prodDLB and DLB groups, Fig. 3A (p < 0.001), and the AUC was 0.875 (95% CI 0.806–0.927) comparing control and prodDLB subjects and 0.944 (95% CI 0.895–0.974), comparing control and DLB patients. CSF NfL levels were elevated in DLB patients with AD pathology (DLB-AD) compared to patients with pure DLB (p = 0.020), Fig. 3B. The AUC for NfL remained lower than the AUC for core AD biomarkers to discriminate these subgroups (data not shown).

#### Down syndrome

**Figure 4:**
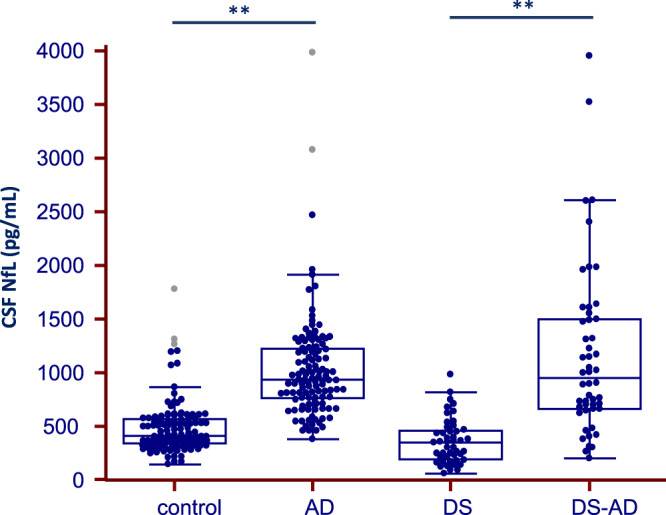
CSF Neurofilament light (NfL) protein levels in patients with Down Syndrome (DS). Box and whisker plots of the median concentrations of CSF NfL in control participants and patients with dementia (Alzheimer Disease, AD) and Down Syndrome associated or not to AD (DS-AD and DS, respectively). The central black lines show the median values, regions above and below these lines show the upper and lower quartiles, respectively. Outliers (indicated with grey circles) are defined as a value that is larger than the upper quartile plus three times the interquartile range. **p < 0.001.

As previously described^18^, CSF NfL levels were increased in the DS-AD group compared to DS group (p < 0.001), Fig. 4 and Table 1. Interestingly, CSF NfL levels were comparable between DS-AD and sporadic AD groups, despite the age difference between groups, Fig. 4.

## Discussion

In the present study, we extend previously published results that highlight the importance of CSF NfL in the evaluation of neurodegenerative diseases^6,9–11,13–16,18,19^. We confirm that ALS and FTD-related syndromes show the highest CSF NfL levels followed by AD and DLB. We also confirm the positive correlation between CSF NfL and age, its association with gender (higher in male)^19^ and its negative correlation with MMSE in various contexts, including control participants, AD and FTD patients^16^. Finally, we report that in DLB patients, CSF NfL levels are influenced by the existence of comorbid AD.

Our results confirm that CSF NfL levels are increased in all neurodegenerative conditions studied compared to control participants^16,19^. In agreement with a recent meta-analysis that included various neurological conditions, our study confirms the overlap of CSF NfL level between various clinical conditions^19^, which may limit its use as a diagnostic marker in the clinical routine of cognitive impairment. In line with other studies^16,20^, we found that ALS patients showed the highest CSF NfL levels. As ALS and FTD are associated in a proportion of patients, we evaluated the differential CSF levels of NfL in ALS, ALS-FTD and FTD. Our results show that CSF NfL levels were increased in the three groups compared to controls, with the highest levels for ALS, followed by ALS-FTD and FTD, in agreement with previously published data^16^. These results are discordant with the recent meta-analysis of Bridel *et al*. describing the ALS-FTD group to be the clinical group with the highest CSF NfL level^19^. This discrepancy may be related to the variability of CSF NfL values in these clinical groups or to the differences in sample size. Although our results suggest that high CSF levels of NfL may be indicative of ALS in the context of FTD, the ROC curves showed moderate diagnostic value and its implementation in clinical routine would therefore require further confirmation. Future studies are needed to determine whether longitudinal changes in CSF NfL meaurements are useful to predict the development of motor neuron disease in patients with FTD. We also found increased CSF NfL levels in patients with CBS and PSP compared to controls, which is in agreement with previous studies^6,9,16,21^. We did not find correlation between MMSE scores and CSF NfL in these two groups, similarly to previously published results^16^. However, such results may be due to the low number of patients in each group (26 and 12, respectively) or to the lack of sensitivity of MMSE to capture cognitive impairment in these disorders.

We also report high CSF NfL levels in patients with DLB compared to controls, in accordance with a previous study^19^. Interestingly, patients with prodDLB within this group showed higher levels of CSF NfL compared to controls. Furthermore, patients in the dementia stage had higher levels compared to prodDLB, while levels of t-Tau, p-Tau and Aβ_1–42_ were similar between both groups. Thus, our results illustrate that CSF NfL levels increase early in DLB, even at prodromal stages, with a further increase in dementia stages. CSF NfL may be of potential value to diagnose prodDLB, as its diagnostic performance appeared higher than CSF core AD biomarkers. These promising results should be further confirmed in a larger cohort. In the present work, we also found higher levels of CSF NfL in DLB patients that had AD copathology compared to DLB patients with negative AD biomarkers. However, the potential added value of CSF NfL for AD pathology in the context of DLB was low (AUC < 0.7, data not shown).

In participants with DS, CSF NfL levels were associated with clinical stages. As previously published^18^, we found a clear and progressive increase of CSF NfL in DS patients with prodromal AD and DS-AD compared to asymptomatic DS participants. These results indicate that CSF NfL could be informative for the diagnosis of dementia in this population, where clinical assessment might be complex. Interestingly, despite the difference in age between DS-AD and sporadic AD patients, NfL levels were similar in both groups indicating a comparable degree in neuroaxonal damage in both types of AD. These results, together with the good correlation of CSF NfL levels with those in plasma found in previous studies^18^, highlight the potential of this biomarker in the diagnosis of dementia in the DS population.

Our work also has some limitations. First of all, the study is retrospective and the clinical protocols differed between clinical groups. Second, the work relied on clinical diagnosis and neuropathological confirmation was not available. Third, MMSE was the only cognitive scale included in this study, which may be less sensitive to capture changes in some groups, such as FTD. Finally, some groups were small and results should be validated in larger cohorts.

In summary, the present work confirms the importance of CSF NfL in the evaluation of neurodegenerative diseases. The study highlights the influence of AD co-pathology on the levels of CSF NfL in DS and DLB and shows the potential interest of CSF NfL determination for early detection of DLB, at prodromal stages of the disease. Taken together, our data show that CSF NfL levels could be a useful addition to the core AD biomarkers in the diagnostic evaluation of neurodegenerative conditions.

## Material and Methods

### Study participants and clinical classification

We included 535 subjects from the SPIN cohort^17^ evaluated at the Memory Unit at Hospital de Sant Pau between January 2009 and October 2017. We included the following diagnostic groups: Alzheimer’s Disease (AD, n = 116), Down Syndrome, without or with dementia (DS, n = 47 and DSAD, n = 50, respectively)^18^, dementia with Lewy Bodies (DLB, n = 37)^22^, prodromal DLB (prodDLB, n = 26)^22^, Amyotrophic Lateral Sclerosis (ALS, n = 46), Frontotemporal dementia (FTD, n = 56)^4,20^, corticobasal syndrome (CBS, n = 26), and progressive supranuclear palsy (PSP, n = 12). Cognitively normal control participants (n = 118) were also included in the present study. All controls had normal cognitive scores in the formal neuropsychological evaluation^23^ and normal core CSF AD biomarkers (see^17^ for further details of the SPIN cohort).

All AD patients had abnormal core AD biomarkers (low Aβ_1–42_ and high t-Tau or p-Tau) in the CSF based on previously published cut-offs^24^. FTD patients with an AD CSF profille (low Aβ_1–42_ and high t-Tau or p-Tau) were excluded from the present study.

Classification of DLB patients was made according to previously published data^22^. Briefly, patients with prodromal DLB (prodDLB) met general criteria for mild cognitive impairment^25^ with at least one sign of α-sinucleinopathy (visual hallucinations, parkinsonism, or REM sleep behaviour disorder (RBD))^26–28^ at the time of evaluation and had to meet criteria of probable DLB during the follow up^29^. Patients with DLB met consensus criteria for probable DLB^29^ and were evaluated using a previously reported clinical protocol, as previously described^17^,^22^. DLB patients with suspected AD copathology were defined according to the ratio tTau/Aβ_1–42_ considering values ≥0.52 as indicative of underlying AD pathology^30^. Patients with ALS fulfilled El Escorial revised criteria^31^ for probable, probable laboratory-supported or definite ALS, and were classified as ALS-FTD according to Raskovsky^32^ criteria.

### CSF collection and analysis

CSF was obtained by lumbar puncture as previously described, collected and processed in polypropylene tubes following international recommendations^33^. CSF levels of core AD biomarkers (Aβ_1–42_, t-Tau, and phosphorylated tau) were measured using commercially available kits from FUJIREBIO-EUROPE (INNOTEST TM, catalog numbers Ref 81583 (Aβ1–42), Ref 81579 (total tau) and Ref 81581 (phosphorylated tau)), as previously described and following provider´s instructions. NfL levels were measured using a commercially available ELISA kit (NF-light, UMAN DIAGNOSTICS, Umea, Sweden,) as previously described^4,20^.

### ApoE genotyping

DNA was extracted using standard procedures and *APOE* was genotyped accordingly to previously described methods^34^.

### Statistical analysis

Because biomarker values were non-normally distributed, the nonparametric Kruskal-Wallis test and the post hoc pairwise Mann-Whitney-tests were used to assess differences between groups. Associations of NfL with other biomarkers and with MMSE score were calculated using Spearman rank correlation. All group comparaisons and correlation analysis were age and sex-adjusted. Alpha threshold was set at 0.05 and ccorrection for multiple comparisons was made with the Bonferroni procedure. X^2^ test was used to assess differences in *APOE*ε4 allele frequency among groups. All tests and area under ROC curve (AUC) analysis were performed using MEDCALC (MEDCALC software ver 15.2.2).

### Ethical approval and consent to participate

The study was approved by the Sant Pau Ethics Committee following the standards for medical research in humans recommended by the Declaration of Helsinki and reported to the Minister of Justice according to the Spanish law for research in people with intellectual disabilities. The protocol of the SPIN cohort was approved by the Sant Pau Ethics Committee. All participants and their legally authorised representative gave written informed consent before enrolment; all controls gave written informed consent before enrolment for their medical information to be used for purposes of scientific research in accordance with the guidelines of the local ethics committee.
